# The influence of process parameters and pulse ratio of precursors on the characteristics of La_1 − *x*_Al_*x*_O_3_ films deposited by atomic layer deposition

**DOI:** 10.1186/s11671-015-0883-6

**Published:** 2015-04-14

**Authors:** Chenxi Fei, Hongxia Liu, Xing Wang, Xiaojiao Fan

**Affiliations:** Key Laboratory for Wide-Band Gap Semiconductor Materials and Devices of Education, School of Microelectronics, Xidian University, No. 2, South Taibai Road, Yanta Zone, Xi’an City, Shaanxi Province 710071 People’s Republic of China

**Keywords:** 81.15−z; 82.80.Pv; 68.37.Ps, Processing parameters, La_2_O_3_, ALD, Dielectric constant, EOT

## Abstract

The influence of processing parameters of aluminum oxide (Al_2_O_3_) and lanthanum oxide (La_2_O_3_) gate dielectric is investigated. Trimethylaluminum (TMA) and tris(isopropylcyclopentadienyl) lanthanum [La(iPrCp)_3_] were used as precursors separately, and H_2_O was used as oxidant. The ultra-thin La_1 − *x*_Al_*x*_O_3_ gate dielectric films are deposited on p-type silicon substrates by atom layer deposition (ALD) for different pulse ratios of precursors. Effects of different La/Al precursor pulse ratios on the physical properties and electrical characteristics of La_1 − *x*_Al_*x*_O_3_ films are studied. The preliminary testing results indicate that the increase of La precursor pulse can improve the characteristics of film, which has significant effects on the dielectric constant, equivalent oxide thickness (EOT), electrical properties, and stability of film.

## Background

It is well known that the key element enabling the successful scaling of Si-based metal-oxide-semiconductor field-effect transistors (MOSFETs) over the last several decades is the traditional gate dielectric, silicon dioxide (SiO_2_), with superior material properties. The miniaturization of MOSFET technology has pushed the conventional SiO_2_ gate dielectric approach to its physical limit [1]. As the thickness of SiO_2_ gate dielectric decreases, it becomes more difficult to grow the superior quality oxides, because excessive tunneling and reliability of ultra-thin SiO_2_ will cause unacceptably high leakage current and degrade the device performance and reliability seriously. Therefore, in order to solve these problems, high-*k* gate dielectrics are being widely considered to replace SiO_2_ [2]. For the past years, people have been attracted by many promising candidates, such as aluminum oxide (Al_2_O_3_), hafnium oxide (HfO_2_), zirconium oxide (ZrO_2_), lanthanum oxide (La_2_O_3_), yttrium oxide (Y_2_O_3_), and their pseudobinary [3-10], and some exhilarating achievements have been obtained. As a compound of La_2_O_3_ and Al_2_O_3_, due to its high dielectric constant (approximately 25), wide energy band gap (5 ~ 6 eV), and thermal stability up to 2,100°C, LaAlO_3_ is considered as a most promising candidate for SiO_2_ replacement [11,12].

LaAlO_3_ films have been deposited on Si substrate using metal-organic chemical vapor deposition (MOCVD), molecular beam epitaxy (MBE), evaporation technique, and pulsed laser deposition (PLD) [12,13]. In contrast to these techniques, atom layer deposition (ALD) is based on self-limiting film growth via alternate saturative surface reaction. Therefore, the films deposited by ALD have high purity, density and accurate thickness. Furthermore, compared to traditional techniques, the deposition temperature can be set between 200°C and 400°C by ALD; this conforms to the future trend of semiconductor industry [14-16]. Due to these incomparable superiorities, LaAlO_3_ films deposited by ALD are widely used in recent years and some marked achievements have been obtained. It is known that the processing parameters determine the quality of films. However, the methods about optimizing each processing parameter have rarely been discussed. In this work, we focus on the influence of processing parameters on the growth of La_2_O_3_ gate dielectric, each processing parameter has been optimized, and the optimal processing parameters are provided for La_2_O_3_ film. Moreover, the La_2_O_3_ and different pulse ratios of La_1 − *x*_Al_*x*_O_3_ films were deposited, and the dielectric constant, equivalent oxide thickness (EOT), and electrical properties of the films are analyzed.

## Methods

La_2_O_3_ and La_1 − *x*_Al_*x*_O_3_ gate stacks were deposited on p-type Si (8 to 12 Ω⋅cm) B-doped (100) wafers using an atomic layer deposition reactor (Picosun R-200, Espoo, Finland). Before the deposition, silicon wafers were cleaned in 80°C solution (NH_4_OH:H_2_O_2_:H_2_O = 1:1:5) for 15 min and followed by a 10 min rinse in deionized water [17]. The thickness of native SiO_2_ layer on Si substrates was determined by ellipsometry as 1.5 nm. For ALD film deposition, trimethylaluminum (TMA) and tris(isopropylcyclopentadienyl) lanthanum [La(iPrCp)_3_] were used as precursors, H_2_O was used as oxidant, and ultra-high purity nitrogen (N_2_, 99.999%) was employed as carrier and purge gas. The container of the aluminum precursor is at room temperature, corresponding to a vapor pressure of 10 to 15 hPa. The pulse time, purge time, heating temperature and deposition temperature of La precursor and pulse time, and purge time of oxidant will be determined by the following experiments. The Al-injection, purge, oxidant injection, and purge times were 0.1, 3, 0.1, and 4 s. For each experiment, 100 cycles of La_2_O_3_ and Al_2_O_3_ films are deposited by ALD. Film thicknesses were measured by Woollam M2000D spectroscopic ellipsometry. The capacitance-voltage (*C*-*V*) measurements were carried out using a Keithley 590 C-V analyzer (Keithley Instruments Inc., Cleveland, OH, USA) at 1 MHz, and the diameter of mercury probe is 859 μm. The bonding structures of the films were examined by X-ray photoelectron spectroscopy (XPS). The atomic force microscopy (AFM) of the films was measured by Seiko SPI3800-SPA-400 scanning probe microscope (Seiko Instruments Inc., Chiba, Japan).

## Results and discussion

### Optimization of heating temperature of La precursor

The heating temperature of precursor is changed from 120°C to 180°C. The La-injection, purge, oxidant injection, and purge times are 0.5, 6, 0.5, and 10 s, and the deposition temperature of films is 300°C.

**Figure 1:**
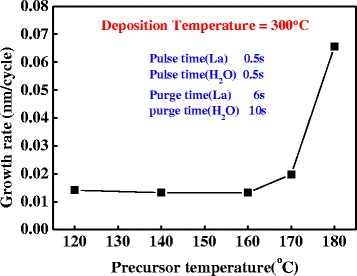
Dependence of the deposition rate of La_2_O_3_ on the precursor heating temperature.

Heating temperature of precursor is an important processing parameter for ALD process. Each precursor has a different boiling point. If the heating temperature of precursor is below the boiling point of precursor, the ALD process cannot operate normally. The heating temperature must be controlled strictly so that the precursor can vaporize. The dependence of the growth rate on the heating temperature of precursor is shown in Figure 1. The growth rate increases with the heating temperature of precursor. When the temperature is below 170°C, the thickness of the deposition films equals the thickness of the natural growth SiO_2_ on Si substrate. This depends on the heating temperature is too low for the La precursor to vaporize. The growth rate is 0.65 nm per cycle when the temperature is 180°C; the experimental growth rate is close to the growth rate reported in previous work [18]. The La precursor can be vaporized when the heating temperature of precursor is above 180°C. Therefore, the best heating temperature can defined as 180°C.

### Optimization of deposition temperature region for La_1 − *x*_Al_*x*_O_3_

Typically, ideal processing parameters of ALD just can operate in a definite range. Under a low deposition temperature, the precursor will stick to the substrate surface when the following precursor reacts to it. The residues of previous substrate exhaust incompletely from the reactor chamber, which causes the increase of the growth rate. On the contrary, in a higher deposition temperature circumstances, the stick precursor can obtain higher energy and can be separated from the surface. The precursor cannot reach saturation adsorption, which will decrease the growth rate of films.

**Figure 2:**
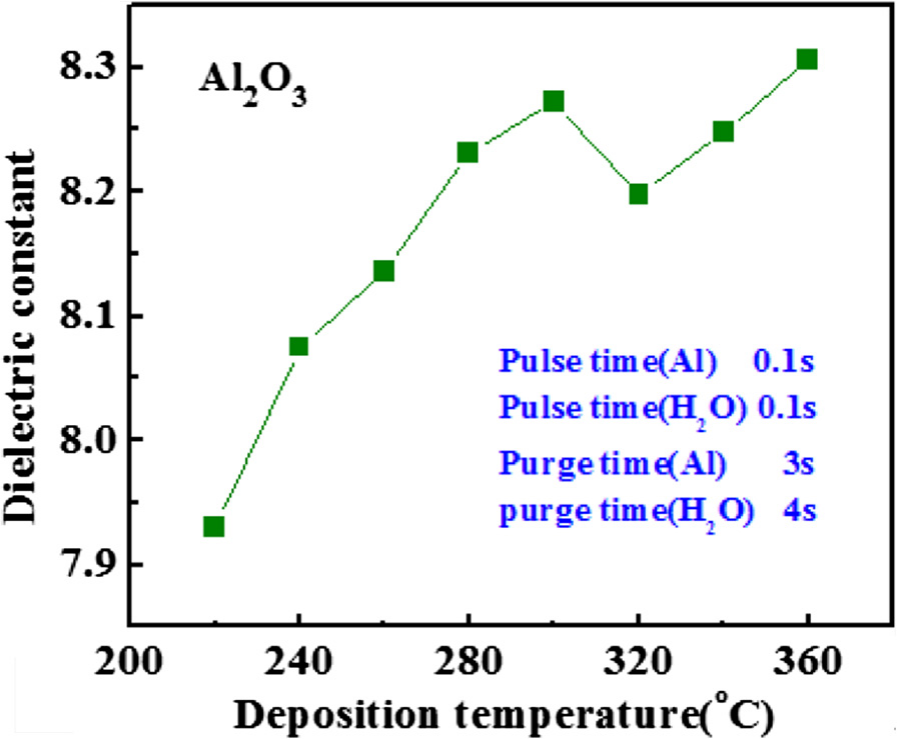
Dependence of the *k* value of Al_2_O_3_ on the deposition temperature.

Figure 2 shows the dielectric constant (*k* value) of Al_2_O_3_ film variation for deposition temperature. The deposition temperature of Al precursor is changed from 220°C to 360°C. The *k* value of Al_2_O_3_ films varies slightly in the range of 220°C to 360°C; the *k* value fluctuates between 7.8 and 8.5. This indicates that the range of deposition temperature fits for the deposition of Al_2_O_3_ films.

**Figure 3:**
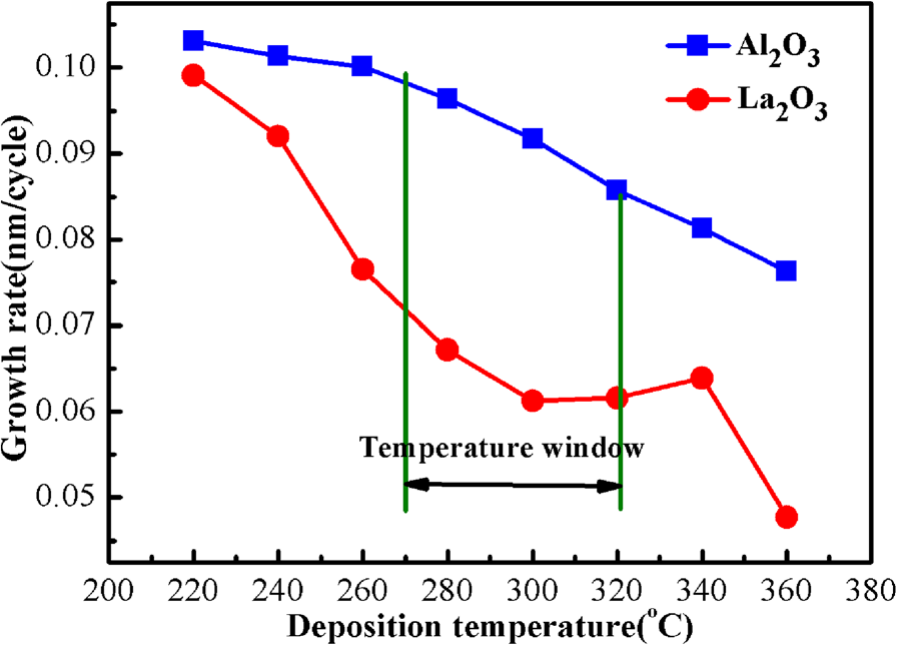
Dependence of the deposition rate of Al_2_O_3_ and La_2_O_3_ on the deposition temperature.

Figure 3 shows the growth rate of Al_2_O_3_ and La_2_O_3_ film variations for deposition temperature. When the temperature is 220°C and 240°C, the growth rate of La_2_O_3_ films is 0.1 and 0.09 nm per cycle. This due to films absorbs many residues, which causes the growth rate is higher than the growth rate of La_2_O_3_ films reported before. On the contrary, the precursors obtain higher energy and separated from the surface when the temperature above 320°C. In this situation, the growth rate of La_2_O_3_ films is only 0.45 nm per cycle, which is lower than the reported data. The growth rate of La_2_O_3_ films is stable when the temperature is between 270°C and 320°C, and the magnitude of growth rate is 0.6 to 0.7 nm per cycle. It proves that the La precursor and the oxidant react completely. So the best deposition temperature range of La_2_O_3_ films is 270°C to 320°C.

Furthermore, in Figure 3, the growth rate of Al_2_O_3_ films reaches the maximum (0.103 nm/cycle) when the deposition temperature is 220°C. The growth rate of Al_2_O_3_ films decreases with increasing deposition temperature. Al_2_O_3_ films have stable growth rate when the deposition temperature is between 270°C and 320°C. So the best temperature window is between 270°C and 320°C for deposition of La_1 − *x*_Al_*x*_O_3_.

### Optimization of pulse and purge time for oxidant of La_2_O_3_ film

The difference of pulse time and purge time also influences the characteristics of high-*k* films. The value of pulse time indicates the quantity of reactant per cycle. The longer pulse time lasts, the more precursors enter reactor chamber. The long pulse time wastes precursors and more precursors stick to the surface, which influences the reaction of precursor in the next cycle. On the other hand, the short pulse time causes incomplete reaction between precursor and groups on surface of substrate, which result in the poor quality and uniform of films.

The numerical value of purge time indicates the quantity of inert gases that enter the reactor chamber per cycle. Shorter purge time cannot clean up the by-product completely, which causes massive by-product remained, such as hydrogen ions, carbon group, and so on. In contrast, longer purge time can introduce other impurities such as moisture, which results in poor efficiency for the growth of materials. Therefore, the reasonable pulse time and purge time is very important for ALD process.

**Figure 4:**
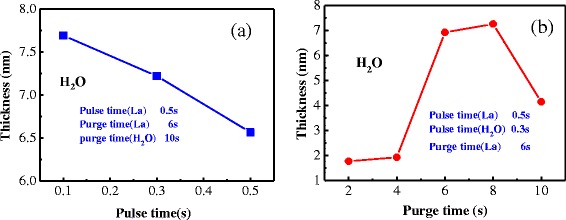
Dependence of the thickness on the pulse and purge time of oxidant. (**a**) Pulse time. (**b**) Purge time.

Figure 4a shows the thickness of La_2_O_3_ film variation with pulse time of oxidant. Heating temperature of La precursor is selected as 180°C, and deposition temperature is selected as 280°C. Pulse/purge time of La precursor is 0.5 s/6 s, and purge time of oxidant is 10 s. Pulse time of oxidant is selected as 0.1, 0.3, and 0.5 s, respectively. The thickness of La_2_O_3_ films decreases with increasing pulse time of oxidant. Because of hygroscopicity of the La_2_O_3_ film, the reaction process can produce carbohydrate and hydroxide, which possess the low dielectric constant. The growth rate of La_2_O_3_ films (0.065 nm per cycle) is close to the reported data when the pulse time is 0.3 s. As the increase of pulse time, the process can produce other by-products. In order to reduce the extent of moisture absorption of La_2_O_3_ films and prevent the production of other by-products, the best purge time for oxidant is 0.3 s.

Figure 4b shows the thickness of La_2_O_3_ film variation with purge time of oxidant. Pulse time of oxidant is 0.3 s; purge time of oxidant is selected as 2, 4, 6, 8, and 10 s, respectively. The thickness of films is almost equal to the thickness of natural growth SiO_2_ when the purge time of oxidant is less than 5 s. In this situation, the La_2_O_3_ films cannot be deposited normally. When the purge time of oxidant is 10 s, the thickness of La_2_O_3_ films is unsatisfactory. It is attributed to the moisture and impurity which injected by long-time nitrogen. The moisture and impurity can damage the deposition of films. Growth rate of La_2_O_3_ films is ideal when purge time takes 6 and 8 s, respectively.

### Optimization of pulse and purge time for La precursor of La_2_O_3_ film

**Figure 5:**
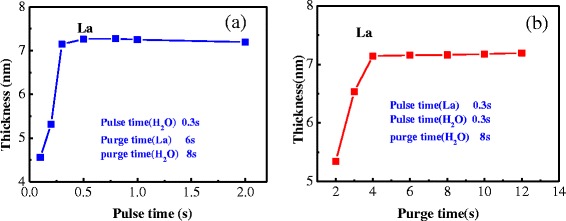
Dependence of the thickness on the pulse and purge time of La precursor. (**a**) Pulse time. (**b**) Purge time.

Figure 5a shows the thickness of La_2_O_3_ film variation with pulse time of La precursor. Pulse/purge time of oxidant is 0.3 s/8 s, and purge time of La precursor is 6 s. Pulse time of La precursor is selected as 0.1, 0.2, 0.3, 0.5, 0.8, 1, and 2 s, respectively. Growth rate of films is unsatisfactory when pulse time of La precursor is less than 0.3 s. Under this circumstance, La_2_O_3_ films cannot deposit completely. Growth rate of films is equal to 0.07 nm per cycle when pulse time of La precursor is over 0.3 s. La_2_O_3_ films reached saturated reaction in the range of time, so the best purge time of La precursor is 0.3 s.

Figure 5b shows the thickness of La_2_O_3_ film variation with purge time of La precursor. Pulse time of La precursor is 0.3 s; purge time of La precursor is selected as 2, 3, 4, 6, 8, 10, and 12 s, respectively. Because of the time interval between two pulses of precursor is very short, residues of previous precursor and by-product produced by reaction cannot be purged from reactor chamber by N_2_. The next pulse of precursor is injected into the reactor chamber, which causes the impurities remain on films, and the deposition is unsatisfactory when the purge time of La precursor is less than 4 s. As purge time is increased, growth rate of films grows stable and ideal. Growth rate of films is better when purge time of La precursor is set at 4 s.

According to the above experiments, the best processing parameters for the deposition of La_1 − *x*_Al_*x*_O_3_ are obtained, which are shown in Table 1.

**Table 1:** **The optimized process parameters of deposition for La**_**1 −*x***_ **Al**_***x***_ **O**_**3**_ **film**

	**Precursor**	**Deposition**	**Precursor**	**Oxidant**	**Precursor**	**Oxidant**
	**temperature (°C)**	**temperature (°C)**	**pulse time (s)**	**pulse time (s)**	**purge time (s)**	**purge time (s)**
La	180	280	0.3	0.3	4	8
Al	25		0.1	0.1	3	4

### Deposition and analysis of La_2_O_3_ and La_1 − *x*_Al_*x*_O_3_ films

In order to research the influence of pulse ratio of precursors on the characteristics of La_1 − *x*_Al_*x*_O_3_ films deposited by ALD, following experiments are designed. The process conditions and thickness of samples are shown in Table 2. Change for deposition cycles guarantees the same thickness for films. Moreover, in order to reduce the influence on the characteristic of films by hygroscopicity, 5 cycles of Al_2_O_3_ are deposited at the top of films [19].

**Table 2:** **Samples with different La/Al precursor pulse ratios**

**Sample**	**Order and cycles for as-deposited films**	**Thickness (nm)**
A	(Al_2_O_3_/La_2_O_3_)*50+ (Al_2_O_3_)*5	10.469
B	(Al_2_O_3_/2*La_2_O_3_)*35+ (Al_2_O_3_)*5	10.757
C	Al_2_O_3_/3*La_2_O_3_)*27+ (Al_2_O_3_)*5	8.895
D	(La_2_O_3_)*121+ (Al_2_O_3_)*5	8.850

**Figure 6:**
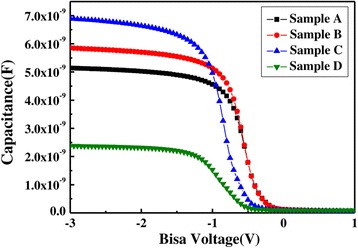
The C-*V* characteristics of the different samples.

*C*-*V* characteristics acquired for samples A, B, C, and D are shown in Figure 6, the sweep voltage from −3 to 1 V. For samples A, B, and C, the value of accumulation capacitance increases with increasing the La/Al precursor pulse ratio. For sample D, pure La_2_O_3_ film, it has the smallest accumulation capacitance. This is due to the moisture adsorption on La_2_O_3_ surface and the La(OH)_3_ has generated, which will lead to a decrease in dielectric constant for film. On the contrary, for samples A, B, and C, the LaAlO_3_ films can reduce the extent of formation for La(OH)_3_. The *C*-*V* curves show a smaller shift in the flatband voltage with the ideal *C*-*V* curve for samples A, B, and C. This attributed to the Al_2_O_3_ doped, which can reduce the extent of diffusion for La atoms and Si atoms and the formation of La-silicate. We can infer that the interfacial layer (IL) of La_1 − *x*_Al_*x*_O_3_ films has high quality. In contrast, negative and large shifts in flatband voltage are observed for sample D. This phenomenon is attributed to the diffusion of La atoms and Si atoms and the formation of positive oxide layer charges, which explains that the IL of pure La_2_O_3_ films has bad quality.

**Figure 7:**
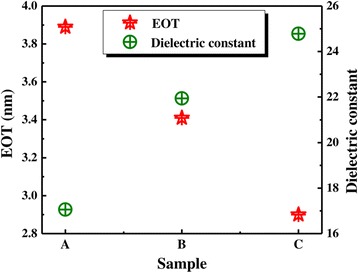
Values of EOT and dielectric constant of the samples.

The extracted dielectric constant and EOT of samples A, B, C, and D are shown in Figure 7. EOT and permittivity are determined as follows:

1 $$ \mathrm{E}\mathrm{O}\mathrm{T}=A\frac{\varepsilon_0{\varepsilon}_{{\mathrm{SiO}}_2}}{C_{\mathrm{ox}}} $$

2 $$ {\mathrm{EOT}}_{\mathrm{high}\hbox{-} k}=\mathrm{E}\mathrm{O}\mathrm{T}-{T}_{{\mathrm{SiO}}_2} $$

3 $$ {\varepsilon}_{\mathrm{high}\hbox{-} k}=\frac{T_{{}_{\mathrm{high}\hbox{-} k}}}{{\mathrm{EOT}}_{{}_{\mathrm{high}\hbox{-} k}}}{\varepsilon}_{{\mathrm{SiO}}_2} $$

where *A* is the area of mercury probe, *C*_ox_ is the accumulation capacitance of films, *T*_SIO2_ is the thickness of native oxide, *ε*_high‐*k*_ is the permittivity values of La_2_O_3_ and La_1 − *x*_Al_*x*_O_3_ films, and ε_SIO2_ and *ε*_0_ are the permittivity values of SiO_2_ and vacuum, respectively [17].

The result indicates EOT decrease and dielectric constant increase with increasing the La/Al precursor pulse ratio for samples A, B, and C. For sample A, EOT and dielectric constant are 3.89 nm and 17.06, respectively; for sample B, EOT and dielectric constant are 3.41 nm and 21.94, respectively; for sample C, EOT and dielectric constant are 2.90 nm and 24.78, respectively. The increase of the dielectric constant is attributed to an increase rate proportional to La, and the theoretical value of dielectric constant (approximately 30) of La_2_O_3_ film is high; this will increase the dielectric constant for La_1 − *x*_Al_*x*_O_3_ films. EOT and dielectric constant for sample D can be calculated in the same way. For sample D, EOT is 8.33 nm and dielectric constant is 5.05. The formation of thicker EOT is caused by generation of the La-silicate which Si atoms from substrate and La atoms from film diffusion. The smaller dielectric constant is caused by the formation of La-hydroxide which is due to the hygroscopicity of La_2_O_3_ films.

**Figure 8:**
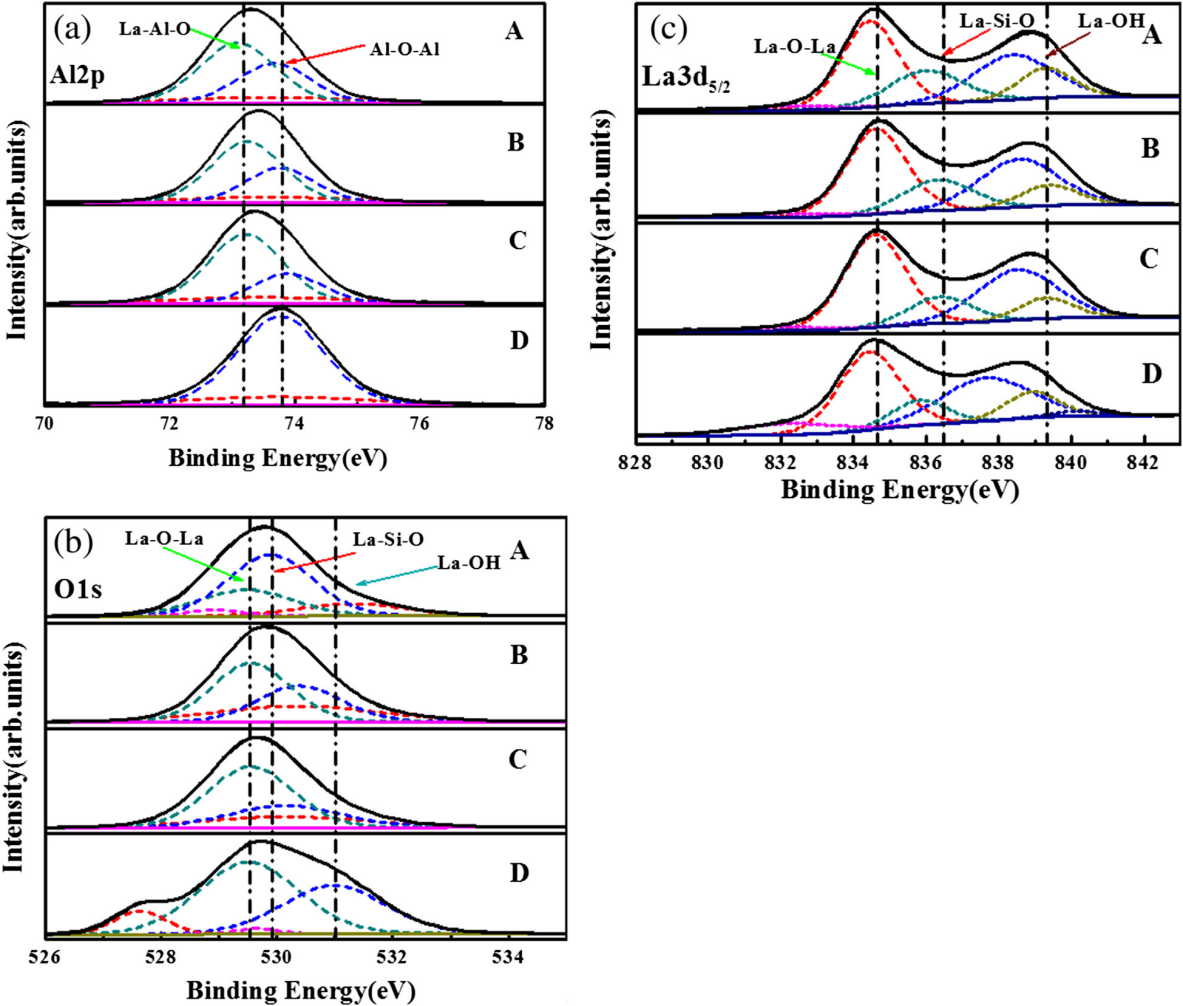
XPS spectra of the different samples. (**a**) Al2p. (**b**) O1s. (**c**) La3d_5/2_.

Figure 8a shows Al2p XPS spectra of the four as-deposited nanolaminate films. The binding energies of core levels are calibrated by setting the adventitious carbon 1-s peak at 284.6 eV [20]. The Al2p spectrum was fitted with three peaks (indicated by dashed line) after the application of a Smart background for samples A, B, and C. The dotted lines indicate the binding energy of La-Al-O and Al-O-Al peaks with the increasing binding energy [19,21]. La-Al-O peak becomes larger and Al-O-Al peak becomes smaller with the increasing La/Al precursor pulse ratio. This indicates La-Al-O bonds combined in sample A more than in sample B and sample C. Moreover, the smallest peaks with red curves are observed, and these peaks probably stand for Al-Si-O and La-Al-Si-O peaks. The formation of Al-Si-O and La-Al-Si-O peaks is caused by the interdiffusion of atoms in Si substrate and as-deposited nanolaminate films. For sample D, the Al2p spectrum was fitted with two peaks, La-Al-O peak and Al-O peak. Al-O peak is attributed to a thin Al_2_O_3_ located at the surface. La-Al-O peak is caused by the contact layer for La_2_O_3_ and Al_2_O_3_ films.

Figure 8b shows O1s XPS spectra of the four as-deposited nanolaminate films. The O1s spectrum was fitted with three peaks (indicated by dashed line) after the application of a Smart background for samples A, B, and C. The dotted lines indicate the binding energy of La-O-La, La-Si-O, and La-OH peaks with the increasing binding energy [22-24]. La-O-La peak becomes larger with the increasing La/Al precursor pulse ratio. Furthermore, smaller La-OH peaks with red curves are obtained. These phenomena are attributed to more combination of La-Al-O bonds and less La-hydroxide, which could make films with good quality. La-Si-O peaks with blue curves become smaller with the increasing La/Al precursor pulse ratio. The formation of La-Si-O peak is caused by the interdiffusion of atoms in Si substrate and as-deposited nanolaminate films. On the other hand, sample D has large La-OH and La-Si-O peaks, and this is caused by the hygroscopic characteristics of pure La_2_O_3_ film and interdiffusion of atoms, respectively.

Figure 8c shows La3d_5/2_ XPS spectra of the four as-deposited nanolaminate films. The dotted lines indicate the binding energy of La-O-La peak, La-Si-O peak, and La-OH peak with the increasing binding energy [20,22,24]. The La-OH peak and La-Si-O peak become smaller with the increasing La/Al precursor pulse ratio. This indicates that the samples have good quality of IL and large dielectric constant, which attribute to the less formation of La-hydroxide and La-silicate. As to sample D, pure La_2_O_3_ films, it has large La-OH peak, and it will cause the reduction of dielectric constant. Moreover, the peak with red curve is observed in sample D, but the binding energy of this peak is smaller than the standard value of La-Si-O peak. We can infer that La-Si-O and La-Al-Si-O were formed in La_2_O_3_ film and attributed to interdiffusion of atoms. These chemical compounds will lead to a reduction of dielectric constant and increase of interfacial layer.

**Figure 9:**
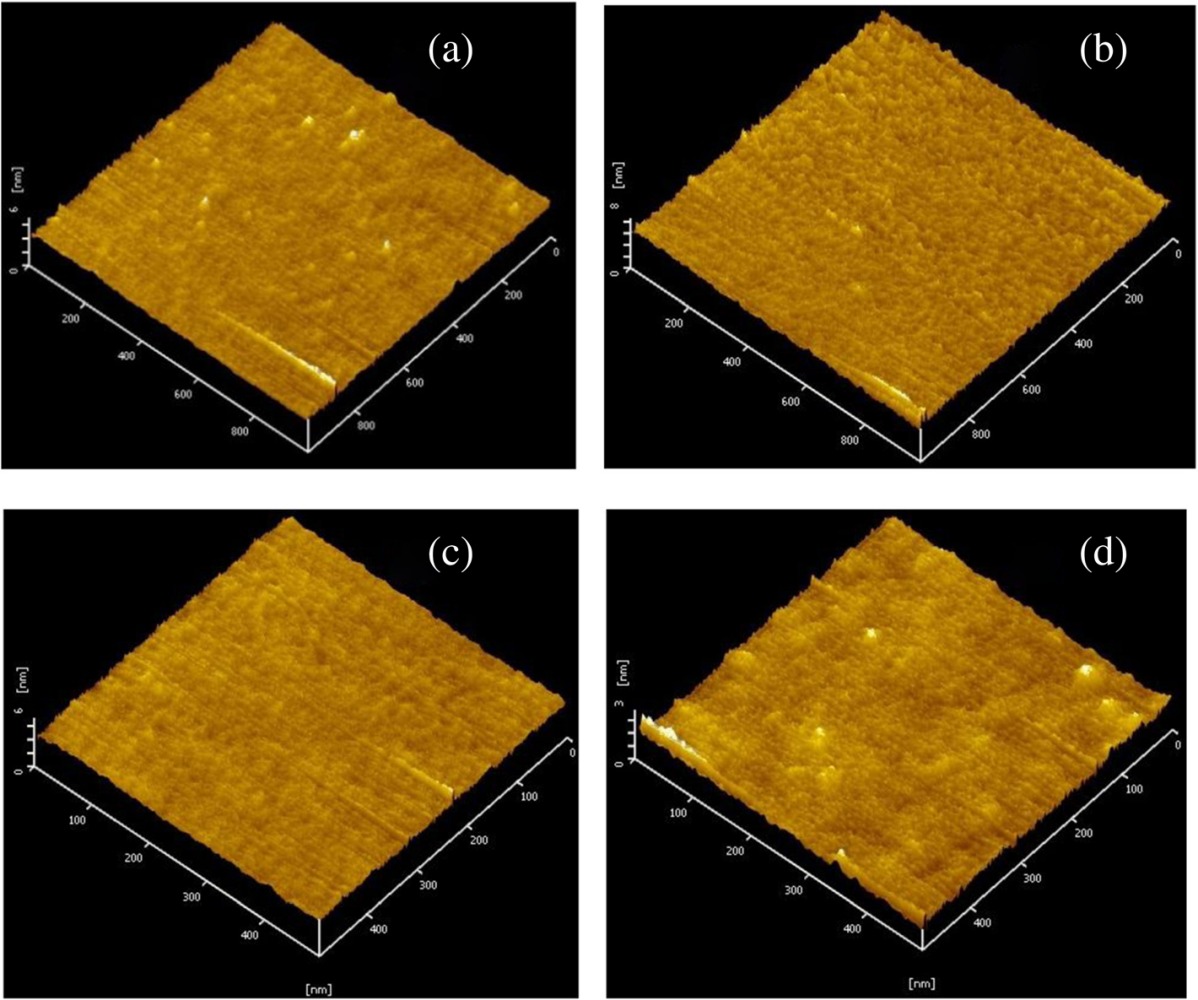
AFM images of sample C and sample D. (**a**) 1 × 1 μm^2^ for sample D. (**b**) 1 × 1 μm^2^ for sample C. (**c**) 0.5 × 0.5 μm^2^ for sample D. (**d**) 0.5 × 0.5 μm^2^ for sample C.

Atomic force microscopy was performed to obtain a three-dimensional image of the surface morphology of films. Figure 9 shows the surface morphology of sample D and sample C. For the typical scan area of 1 × 1 μm^2^, the surface roughness of sample D and sample C was measured at 0.48 and 0.39 nm, respectively; for the typical scan area of 0.5 × 0.5 μm^2^, the surface roughness of sample D and sample C was measured at 0.46 and 0.27 nm, respectively. This indicates that La_2_O_3_ films have rough surface, and it is caused by the hygroscopic characteristics of pure La_2_O_3_ film which formed La-hydroxide [25]. On the contrary, sample C has smooth surface due to the stable characteristic for La_1 − *x*_Al_*x*_O_3_ film. The results are in accordance with the XPS analysis results above.

**Figure 10:**
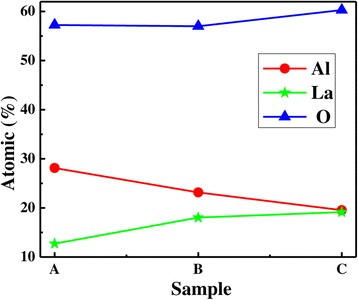
Percentage compositions of La, Al, and O atoms in different samples.

Table 3 and Figure 10 show the percentage compositions of different atoms in samples A, B, C, and D. The percentage composition of La atoms in films increases with increasing La/Al precursor pulse ratio. The percentage composition of La is 12.72% and Al is 28.12% for sample A, and the ratio of La:Al is approximately 1:2; the percentage composition of La is 18.04% and Al is 23.16% for sample B, and the ratio of La:Al is approximately 1:1.3. However, the percentage composition of La is 19.16% and Al is 19.54% for sample C, and the ratio of La:Al is approximately 1:1. It indicates that a part of La atoms diffuse from the substrate and form La-silicate when the La/Al precursor pulse ratio is low, and a small part of La atoms is attributed to the formation of La-Al-O bonds. The large part of La atoms is attributed to the formation of La-Al-O bonds with increasing La/Al precursor pulse ratio. The ratio of La:Al:O is approximately 1:1:3 for sample C. This result is close to the standard element stoichiometry for LaAlO_3_ films, which also validate the results of XPS. For sample D, the proportion of La is 32.79% and O is 56.62%, and the ratio of La:O is approximately 1:1.7. The phenomenon of O-rich is attributed to formation of Al_2_O_3_, La-hydroxide, and La-silicate.

**Table 3:** **Percentage compositions of different atoms in samples**

	**Al2p (at%)**	**La3d (at%)**	**O1s (at%)**	**C1s (at%)**	**N1s (at%)**
Sample A	28.12	12.73	57.25	1.48	0.42
Sample B	23.16	18.04	57.01	0.82	0.97
Sample C	19.16	19.54	60.30	0.94	0.36
Sample D	8.80	32.79	56.62	0.83	0.96

According to the ratio of La to Al analyzed by XPS, we can observe that the content of La element is insufficient in La_1 − *x*_Al_*x*_O_3_ films. La_1 − *x*_Al_*x*_O_3_ film is preserved in air for a long time after deposition, which prohibit La and Al atoms diffuse from the substrate and form interfacial layer. Moreover, diffusion coefficient of Al element is higher than that of La; diffusion of La element is faster than that of Al. The closer to the Si substrate, the more La atoms lose in the as-deposited films. Therefore, the ratio of La to Al is less than 1. Moreover, the oxygen atoms in films are abundant. The main reason is that the inter gases contain a little moisture, and it will induce the excessive oxygen content.

## Conclusions

In this study, we mainly investigated that the influence of process parameters and pulse ratio of precursors on the characteristics of La_1 − *x*_Al_*x*_O_3_ films is deposited by ALD. Firstly, main process parameters for ALD which include heating temperature of precursor, deposition temperature window, pulse time, and purge time for precursor and oxidant, respectively, are obtained. The optimized process parameters of La_1 − *x*_Al_*x*_O_3_ film are presented. Furthermore, as the increase of the La/Al precursor pulse ratio, more La-Al-O bonds were formed in La_1 − *x*_Al_*x*_O_3_ films. When the ratio of La and Al precursor is 3 to 1, the film has small EOT, high dielectric constant, excellent interface layer, and superior character of surface. XPS analysis shows that the La/Al/O ratio of sample with a La/Al precursor pulse ratio of 3/1 fits the stoichiometric LaAlO_3_ composition (1:1:3) better, and this result is close to the standard value, thereby making them become a suitable insulator in MIM capacitor devices.

## References

[1] Essa Z, Gaumer C, Pakfar A, Gros-Jean M, Juhel M, Panciera F (2012). Evaluation and modeling of lanthanum diffusion in TiN/La_2_O_3_/HfSiON/SiO_2_/Si high-k stacks. Appl Phys Lett.

[2] Jin H, Cho YJ, Oh SK, Kang HJ, Park JC, Heo S (2008). Band gap engineering for La aluminate dielectrics on Si (100). Appl Phys Lett.

[3] Poodt P, Lankhorst A, Roozeboom F, Spee K, Maas D, Vermeer (2010). High-speed spatial atomic-layer deposition of aluminum oxide layers for solar cell passivation. Adv Mater.

[4] Fan JB, Liu HX, Gao B, Ma F, Zhuo QQ, Hao Y (2012). Influence of different oxidants on the band alignment of HfO_2_ films deposited by atomic layer deposition. Chin Phys B.

[5] Jaakko N, Miia M, Kaupo K, Leila C, Esa P, Mikko R (2010). Growth and phase stabilization of HfO_2_ thin films by ALD using novel precursors. J Cryst Growth.

[6] Nam WH, Rhee SW (2004). Atomic layer deposition of ZrO_2_ thin films using dichlorobis[bis-(trimethylsilyl)amido]zirconium and water. Chem Vap Deposition.

[7] Chen CH, Chang IYK, Lee JYM, Chiu FC, Chiou YK, Wu TB (2007). Reliability properties of metal-oxide-semiconductor capacitors using LaAlO_3_ high-k dielectric. Appl Phys Lett.

[8] Chiu FC, Chou HW, Lee JY (2005). Electrical conduction mechanisms of metal/La_2_O_3_/Si structure. J Appl Phys.

[9] Hsu CH, Wang MT, Lee JYM (2006). Electrical characteristics and reliability properties of metal-oxide-semiconductor field-effect transistors with La_2_O_3_ gate dielectric. J Appl Phys.

[10] Chu RL, Chiang TH, Hsueh WJ, Chen KH, Lin KY, Brown GJ (2014). Passivation of GaSb using molecular beam epitaxy Y_2_O_3_ to achieve low interfacial trap density and high-performance self-aligned inversion-channel p-metal-oxide-semiconductor field-effect-transistors. Appl Phys Lett.

[11] Suzuki M, Yamaguchi T, Fukushima N, Koyama M (2008). LaAlO_3_ gate dielectric with ultrathin equivalent oxide thickness and ultralow leakage current directly deposited on Si substrate. J Appl Phys.

[12] Becerra L, Merckling C, Baboux N, Plossu C, Marty O, El-Kazzi M (2007). Ultralow equivalent oxide thickness obtained for thin amorphous LaAlO_3_ layers grown on Si(001). Appl Phys Lett.

[13] Shao QY, Li AD, Cheng JB, Ling HQ, Wu D, Liu ZG (2005). Growth behavior of high k LaAlO_3_ films on Si by metalorganic chemical vapor deposition for alternative gate dielectric application. Appl Surf Sci.

[14] Niinistö J, Kukli K, Heikkilä M, Ritala M, Leskelä M (2009). Atomic layer deposition of high-*k* oxides of the group 4 metals for memory applications. Adv Eng Mater.

[15] Niinistö L, Nieminen M, Päiväsaari J, Niinistö J, Putkonen M, Nieminen M (2004). Advanced electronic and optoelectronic materials by atomic layer deposition: an overview with special emphasis on recent progress in processing of high-k dielectrics and other oxide materials. Phys Stat Sol.

[16] Miikkulainen V, Leskelä M, Ritala M, Puurunen RL (2013). Crystallinity of inorganic films grown by atomic layer deposition: overview and general trends. J Appl Phys.

[17] Fan JB, Liu HX, Ma F, Zhuo QQ, Hao Y (2013). Influences of different oxidants on the characteristics of HfAlO_*x*_ films deposited by atomic layer deposition. Chin Phys B.

[18] Kim WS, Park SK, Moon DY, Kang BW, Kim HD, Park JW (2009). Characteristics of La_2_O_3_ thin films deposited using the ECR atomic layer deposition method. J Korean Phys Soc.

[19] Liu JW, Liao MY, Imura M, Oosato H, Watanabe E, Tanaka A (2013). Interfacial band configuration and electrical properties of LaAlO_3_/Al_2_O_3_/hydrogenated diamond metal-oxide-semiconductor field effect transistors. J Appl Phys.

[20] Ai-Dong L, Qi-Yue S, Hui-Qin L, Jin-Bo C, Di W, Zhi-Guo L (2003). Characteristics of LaAlO_3_ gate dielectrics on Si grown by metalorganic chemical vapor deposition. Appl Phys Lett.

[21] McDonnell S, Pirkle A, Kim J, Colombo L, Wallace RM (2012). Trimethyl-aluminum and ozone interactions with graphite in atomic layer deposition of Al_2_O_3_. J Appl Phys.

[22] Maeng WJ, Woo-Hee K, Hyungjun K (2010). Flat band voltage modulation by controlling compositional depth profile in nanolaminate gate oxide. J Appl Phys.

[23] Kim J, Kim H, Wallace RM, Park TJ (2012). In-situ XPS study on ALD (atomic layer deposition) of high-k dielectrics: La_2_O_3_ using La-formidinate and ozone. ECS Trans.

[24] Wong H, Zhou J, Zhang J, Jin H, Kakushima K, Iwai H (2014). The interfaces of lanthanum oxide-based subnanometer EOT gate dielectrics. Nanoscale Res Lett.

[25] Zhao Y, Toyama M, Kita K, Kyuno K, Torium A (2006). Moisture-absorption-induced permittivity deterioration and surface roughness enhancement of lanthanum oxide films on silicon. Appl Phys Lett.

